# Women’s empowerment and uptake of sulfadoxine–pyrimethamine for intermittent preventive treatment of malaria during pregnancy: results from a cross-sectional baseline survey in the Lake endemic region, Kenya

**DOI:** 10.1186/s12936-023-04679-z

**Published:** 2023-08-23

**Authors:** George Odwe, Dennis Juma Matanda, Tchaiwe Zulu, Stephen Kizito, Oscar Okoth, Beth Kangwana

**Affiliations:** 1 Population Council, Kenya, Avenue 5, 3rd Floor, Rose Avenue, P.O Box 17643-00500, Nairobi, Kenya; 2 Kisumu Medical and Education Trust (KMET), P. O Box 6805-40103, Kisumu, Kenya

**Keywords:** Women empowerment, Intermittent treatment, Sulfadoxine–pyrimethamine, Malaria in pregnancy, Kenya

## Abstract

**Background:**

Malaria in pregnancy remains a major public health problem in endemic areas of the sub-Saharan African (SSA) region. However, there is limited understanding of the association between women’s empowerment and the uptake of sulfadoxine–pyrimethamine for intermittent preventive treatment of malaria during pregnancy (IPTp-SP) in Kenya. This study examines the association between women’s empowerment indicators (decision-making power, control of assets, education, and employment status) and the uptake of three or more doses of IPTp-SP in the Lake endemic region of Kenya.

**Methods:**

The analysis utilized a dataset from a cross-sectional baseline survey of 3129 women aged 15–49 years in Kisumu and Migori Counties who had a live birth within the last 2 years preceding the study. Data were collected between June to August 2021. A descriptive analysis was conducted to show the distribution of respondents by key background characteristics, and bivariate and multivariate logistic regression to examine statistically significant associations between women’s empowerment measures and the uptake of 3+ doses of IPTp-SP.

**Results:**

Among the 3129 women surveyed, 1978 (65.7%) received 3+ doses of IPTp-SP during their most recent pregnancy. Controlling for individual characteristics and the number of ANC visits, the odds of taking 3+ doses of IPTp-SP increased among women who had high decision-making autonomy (AOR = 2.33; CI = 1.81–3.01; P < 0.001); and tertiary level of educational attainment (AOR = 1.51; CI = 1.10–2.06). However, the association between control of assets and uptake of IPTp-SP was positive but not statistically significant.

**Conclusion:**

Women’s decision-making autonomy and educational attainment were positively associated with the uptake of IPTp-SP. As a result, maternal health interventions should focus on less empowered women, specifically those with less decision-making autonomy and no/low formal education, as they are less likely to achieve optimal uptake of IPTp-SP during pregnancy.

## Background

In 2021, there were approximately 247 million malaria cases globally, with the sub-Saharan Africa (SSA) region bearing the heaviest burden, accounting for about 95% of cases and 96% of malaria-related deaths [1]. Kenya, in particular, faces significant malaria risk, with about 70% of the population at risk, including 13 million in endemic areas and 19 million in highland epidemic-prone and seasonal transmission areas [2, 3]. Malaria is a leading cause of morbidity and mortality in the country, responsible for nearly a third (31%) of outpatient cases according to estimates from the Ministry of Health (MOH) [4]. Pregnant women are especially vulnerable: about 1.8 million pregnant women in Kenya are at risk of malaria infection [5]. Malaria in pregnancy (MiP) is a common cause of spontaneous abortion and has devastating consequences on maternal, newborn, infant, and child health outcomes, including severe maternal anaemia, low infant birth weight, neonatal mortality, preterm delivery, and stillbirth [6].

The World Health Organization (WHO) recommends intermittent preventive treatment in pregnancy (IPTp) with sulfadoxine–pyrimethamine (SP) as a malaria preventive chemotherapy [7]. IPTp-SP has been proven to reduce adverse effects of MiP on maternal and fetal outcomes when administered to pregnant women, starting as early as possible in the second trimester, with monthly intervals up to delivery during Antenatal Care (ANC) visits [8, 9]. Kenya adopted WHO’s IPTp-SP strategy and established its first national IPTp policy in 1998, later updated in 2009 and 2012 to focus on moderate to high transmission areas (i.e., endemic lake and coastal regions). The IPTp strategy is integrated into the overall ANC package for maternal health, offering an opportunity to provide the recommended three or more (3+) doses of IPTp-SP [10]. However, the uptake of 3+ doses of IPTp-SP among pregnant women remains below the national target of 80%. According to recent estimates from the 2020 Kenya Malaria Indicator Survey (KMIS), only 22% of women aged 15–49 with a live birth in the past 2 years before the survey completed 3+ doses of IPTp-SP [3].

A vast amount of literature addresses the factors associated with the uptake of 3+ doses of IPTp-SP in SSA. Major strands of evidence indicate that the uptake of 3+ doses of IPTp-SP is associated with the mother’s level of education [10], socio-economic status [11], knowledge about malaria prevention/prophylaxis and treatment [12], as well as the number and timing of ANC visits [12, 13]. Demographic factors such as age, parity, and health system-related factors have also been linked to the uptake of 3+ doses of IPTp-SP [14]. Furthermore, evidence shows that women’s empowerment plays a role in healthcare utilisation, including ANC attendance [15–18]. However, its specific impact on the uptake of 3+ doses of IPTp-SP is less well understood in Kenya [19]. Additionally, the relationship between women’s empowerment and healthcare utilization may vary by context.

The concept of women’s empowerment is multifaceted [20]. It encompasses various dimensions, including women’s decision-making power at the household level, control over resources, and their ability to make effective choices that can positively lead to desired health and education outcome and overall well-being [21]. Other studies have conceptualized women’s empowerment in the form of human capital (measured through educational attainment) and access to economic resources, such as ownership of productive assets like land or employment status [22]. Consequently, solely relying on a single measure of women’s empowerment may not provide a comprehensive understanding when examining associations or establishing targets for health intervention programmes.

This study examines the association between various indicators of women’s empowerment (decision-making power, control of assets, education, and employment status), and the uptake of 3+ doses of IPTp-SP among women aged 15–49 years who had a live birth within the 2 years prior to the survey. The study hypothesis is that women’s empowerment is positively associated with the uptake of 3+ doses of IPTp-SP during their most recent pregnancy. Exploring the association between women’s empowerment and the uptake of 3+ doses of IPTp-SP can provide valuable insights for malaria prevention programmes and inform their strategies effectively.

## Methods

### Study design

The data for this paper are derived from a cross-sectional baseline survey conducted for the Revive IPTp-SP project. This project aims to revitalize and support the effective delivery and uptake of IPTp-SP in Kenya.

### Study sites

The study was conducted in two malaria-endemic counties, namely Migori and Kisumu, situated in the Lake region of Kenya [3]. Both counties experience stable malaria transmission, with altitudes ranging from 0 to 1300 m around Lake Victoria. Malaria transmission in these regions is perennial, influenced by factors like rainfall, temperature, and humidity. The favourable climatic conditions support a short vector life cycle and high survival rate. Malaria transmission is intense throughout the year, with annual entomological inoculation rates between 30 and 100. Migori and Kisumu counties have a combined population of 2.27 million, with approximately 52% being women, and half of them are of reproductive age (15–49 years) [3].

Kenya’s IPTp strategy recommends provision of IPTp-SP at each routine ANC visit under directly observed therapy (DOT), starting as early as possible in the second trimester. However, HIV-positive pregnant women on daily cotrimoxazole chemoprophylaxis are not recommended to receive IPTp-SP for the prevention of MiP [23]. According to estimates from the 2020 KMIS s the uptake of 3+ doses of IPTp-SP in the Lake endemic region (encompassing Kisumu, Migori, and other counties near Lake Victoria) improved from 35% to 2015 to 49% in 2020. Despite the progress, half of the pregnant women in these regions still have sub-optimal uptake of the recommended 3+ doses of IPTp-SP, even with high ANC attendance. In 2022, the proportion of women 15–49 years making 4+ ANC visits was estimated at 63% and 59% for Kisumu and Migori, respectively [24].

### Target population

The target population comprised women aged 15–49 years, who had a pregnancy and gave birth to a live baby within the 2 years preceding the survey and were residents of Kisumu and Migori counties.

### Sample size and sampling

A sample size of 4080 women (2470 in Migori and 1610 in Kisumu) aged 15–49 years, who had a pregnancy and gave birth to a live baby within the last 2 years preceding the survey were targeted for inclusion in the study. The sample size was determined to detect a minimum 5-percentage point increase in the proportion of women taking three or more doses of IPTp-SP at follow-up, with at 95% confidence level and 80% power, while accounting for homogeneity among participants from the same clusters and a 25% non-response rate [25]. To select the sample, the 2019 national census data was used to generate a list of sub-locations (the smallest administrative unit). Forty sub-locations (16 out of 175 in Kisumu and 24 out of 230 in Migori) were randomly sampled from this list. A research team was deployed to conduct a household listing, which served as the sampling frame. A household was considered eligible if it had a woman aged 15–49 who had a pregnancy and gave birth to a live baby within the last 2 years preceding the survey. In households with more than one woman eligible for the interview, only one participant was randomly selected and interviewed.

### Data collection

Data collection occurred from June to August 2021. The research assistants with social science background and prior experience conducting similar surveys, underwent a 5-days training before being deployed to the study sites for data collection. Data were collected electronically using a standardised questionnaire programmed in Open Data Kit (ODK) and administered through Android-enabled tablets. The questionnaire captured various information, including socio-demographic characteristics (e.g., age, level of education, number of births, marital status), decision-making, control of productive assets such as land, uptake of IPTp, administration of IPTp, ANC attendance, malaria morbidity during pregnancy, and knowledge about malaria prevention approaches during pregnancy, among other relevant data.

### Variables

#### Outcome variable

The uptake of 3+ doses of IPTp-SP during the last pregnancy served as the outcome variable. As recommended by the WHO, the uptake of at least three doses of IPTp-SP during pregnancy is a cornerstone for curbing MiP in areas with moderate to high malaria transmission [7]. Details of IPTp-SP uptake were extracted from the ANC clinic attendance booklet. For women without an ANC booklet, they were asked to indicate whether they had taken a medicine called SP to prevent getting malaria during their last pregnancy. If they answered positively, they were further asked to specify the number of times they took SP during their last pregnancy. Women with an ANC attendance card indicating or reporting taking at least three doses of IPTp-SP during their last pregnancy were coded 1, and 0 otherwise.

#### Independent variables

The main independent variables were:

1. Decision-making autonomy: Decision-making autonomy was measured based on, four questions that asked whether the respondent participated in decisions regarding personal earnings, her healthcare, household purchases, and family visits. Each response was dichotomized into 1 (participates in decision-making), and 0 otherwise. The resulting measure was reliable (Cronbach’s alpha = 0.63). Subsequently, a three-category variable was created based on the four binary variables with values 0 representing no/low decision-making power, 1–2 (moderate decision-making power), and 3–4 (high decision-making power). A similar approach have been employed in previous studies to generate a single index for women’s autonomy or empowerment [11, 15].
2. Control over productive assets: This variable was computed based on four questions that asked the respondents regarding land ownership, ownership of productive assets (e.g., animals and machinery), having independent source of income, and making household purchase using their income. The resulting measure was reliable (Cronbach’s alpha = 0.60). Each item was dichotomized into 1 (yes) and 0 (no). Subsequently, a three-category variable out of the four binary variables was created with values 0 indicating no control over productive assets, 1–2 (moderate control over productive assets), and 3–4 (high control over productive assets). Similar measures of women’s empowerment have been proposed in previous studies [26].
3. Educational attainment: Educational attainment was used to capture human capital. This variable was categorized as follows: (1 = No schooling/ some primary, 2 = primary completed and 3 = Secondary completed and 4 = Tertiary).
4. Employment status: Employment status served as a proxy of social independence or skills acquisition and productivity. This variable was derived from women’s self-reports on their economic activity engagement and was coded as (1 = unemployed and 2 = employed (formal or informal).

#### Control variables

Control variables include (1) socio-demographic factors: (a) respondent’s age categorized into three [15–24, 25–34, and 35+ years]; (b) marital status categorized into two [married, not married]; and (c) gravidity categorized into four [one, two, three, four or more]; and (2) knowledge of MiP prevention—a composite variable based on five questions that asked respondents their knowledge of approaches used for preventing MiP (i.e., sleeping under an insecticide-treated bed net, taking SP, indoor residual spraying), gestational age one should start taking IPTp-SP (i.e., between 13 and 16 weeks), and the recommended number of doses of SP that a pregnant woman is expected to take during entire pregnancy period (i.e., three or more doses). Each item was dichotomised into 1 (yes) and 0 (otherwise). Finally, a three-category variable was created out of the five binary variables with values 0–1 (no/low knowledge), 2–3 (moderate knowledge), and 4–5 (high knowledge).

The analysis adjusted for the number of ANC visits as the entry step for women in this study to receive the WHO-recommended intervention package. Women who completed at least four ANC visits were coded 1 and 0 otherwise. The rationale is that women who completed at least four ANC visits should have received at least three doses of IPTp-SP according to the national guidelines for diagnosing, treating, and preventing malaria in Kenya [4, 23].

### Statistical analysis

The analysis involved: (1) descriptive statistics (frequencies) to show the distribution of respondents by key background characteristics and uptake of 3+ doses of IPTp-SP; and (2) bivariate (model I) and multivariate (models II and III) logistic regression to determine the association between women’s empowerment (decision-making power, control over productive assets, education and employment) and the uptake of 3+ doses of IPTp-SP. Model II adjusted for individual characteristics (age, marital status, gravidity and knowledge of MiP prevention) to examine the degree to which the association between women’s empowerment and the uptake of 3+ IPTp-SP doses is affected by current individual attributes. Model III adjusted for the effects of ANC visits to test the robustness of the result to exclusion or inclusion of this variable that acts as the entry point for receiving IPTp-SP via DOT. Comparing models II and III helps avoid making “The Table 2 Fallacy” (i.e., reporting multiple adjusted effect estimates from a single model; see [26]).

**Fig. 1:**
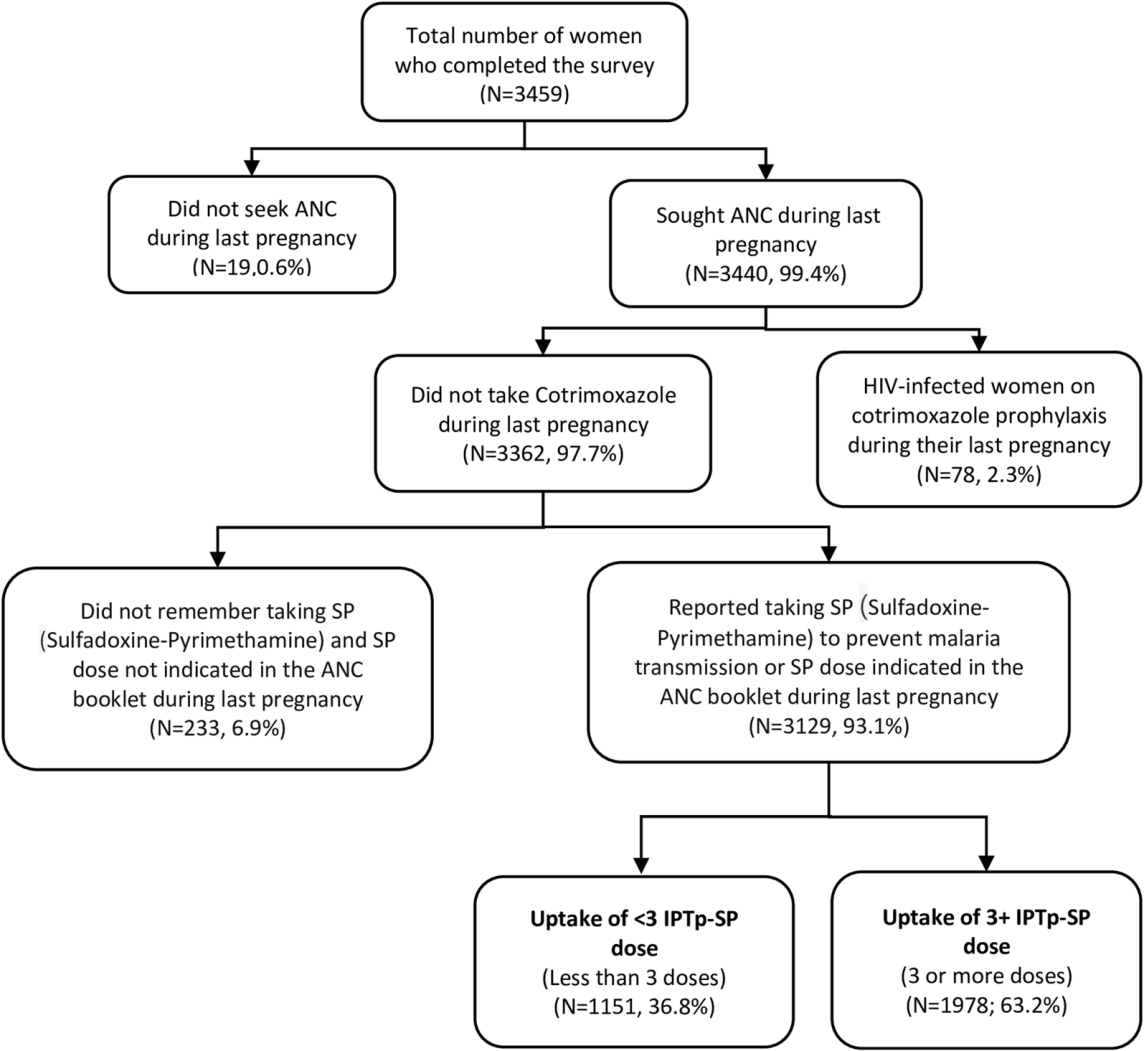
Flow diagram of IPTp-SP uptake among women 15–49 years who had a pregnancy and gave birth to a live baby within the last 2 years preceding the survey in Kisumu and Migori counties, Kenya

Crude odds ratios (c*OR*) and adjusted odds ratios (a*OR*) were reported with 95% confidence intervals (CI), and all predictors with *p* < 0.05 were considered to be independently associated with the uptake of 3+ doses of IPTp-SP. A weighted analysis was performed to account for the complex survey structure and derive representative estimates using the svy command in Stata. Correlation and Variance Inflation Factor (VIF) analyses were performed to check for multicollinearity and tolerance values of all variables. No major multicollinearity problems were found (mean VIF 1.18). Figure 1 represents how the analytical sample was achieved at. The analysis was restricted to women with at least one ANC visit to ensure that only women exposed to WHO-recommended IPTp intervention via DOT were included. In addition, the analysis excluded women who (1) did not have an ANC booklet and could not remember taking SP during their last pregnancy; and (2) were HIV-positive and reported receiving cotrimoxazole prophylaxis. Statistical analyses were performed using Stata version 15.1 (Stata Corp., College Station, TX).

### Ethical considerations

The study received ethical approval from the Population Council’s Institutional Review Board (PC-IRB-Protocol 962) and the AMREF Ethics and Scientific Review Committee (AMREF-ESRC P886/2020). In addition, the Kenya National Commission for Science, Technology, and Innovation (NACOSTI/P/21/8778) granted research clearance. Written informed consent was obtained from participants aged 18 years and above and emancipated minors before conducting the interviews.

## Results

### Background characteristics of the study population

Table 1 presents the background characteristics of the respondents by uptake of IPTp-SP. Out of 3129 women who reported taking SP to prevent malaria transmission or had SP doses indicated in the ANC booklet during their last pregnancy, 1978 (65.7%) completed three or more doses of IPTp-SP, while 1151(34.3%) completed two or fewer doses. The majority of the women were below the age of 35 years (86.6%) and were married (80.4%). Approximately one out of four women (25.1%) had given birth to four or more children, and more than a third (34.1%) had low knowledge of MiP prevention approaches. Moreover, about one-third of the women (33.9%) had completed primary education, 52.9% were employed, 40.2% had a high level of decision-making power, and 55.4% had significant control over assets. Additionally, a large proportion (84.0%) had made four or more ANC visits during their last pregnancy.

**Table 1:** Distribution of respondents by background characteristics and the uptake of IPTp-SP among women who had a live birth in the last 2 years preceding the survey

	IPTp-SP Uptake				All women	
	< 3 doses		3 + doses			
	N^^^	Weighted % [95 CI]	N^^^	Weighted % [95 CI]	N^^^	Weighted % [95 CI]
Age						
15–24	530	42.8 [24.3, 63.6]	820	33.8 [22.3, 47.7]	1350	36.9 [22.7, 53.8]
25–34	474	47.5 [34.5, 60.8]	852	50.9 [43.0, 58.7]	1326	49.7 [39.9, 59.5]
35–49	147	9.7 [4.3, 20.6]	306	15.3 [10.6, 21.5]	453	13.4 [8.1, 21.2]
Marital status						
Not married	210	18.4 [13.8, 24.1]	310	20.2 [12.6, 30.8]	520	19.6 [13.1, 28.3]
Married	941	81.6 [75.9, 86.2]	1668	79.8 [69.2, 87.4]	2609	80.4 [71.7, 86.9]
Gravidity						
One	279	35.4 [29.4, 42.0]	452	24.6 [18.1, 32.6]	731	28.3 [21.9, 35.8]
Two	235	29.7 [22.8, 37.6]	436	26.5 [23.7, 29.6]	671	27.6 [23.6, 32.0]
Three	207	11.9 [6.2, 21.6]	347	22.7 [17.6, 28.8]	554	19.0 [16.8, 21.4]
Four or more	430	23.0 [15.7, 32.4]	743	26.1 [22.2, 30.5]	1,173	25.1 [20.1, 30.8]
Knowledge of MiP prevention						
Low	526	41.2 [33.4, 49.6]	862	40.1 [36.7, 43.6]	1388	40.5 [35.8, 45.3]
Moderate	353	30.7 [25.9, 35.9]	557	22.7 [15.8, 31.4]	910	25.4 [19.2, 32.8]
High	272	28.1 [20.0, 37.9]	559	37.3 [29.9, 45.3]	831	34.1 [26.1, 43.1]
Level of education						
No schooling or some primary	380	13.8 [7.5, 24.1]	576	11.5 [6.5, 19.6]	956	12.3 [6.9, 21.2]
Primary completed	473	3.9 [20.6, 50.3]	801	33.9 [25.9, 42.8]	1274	33.9 [24.3, 44.9]
Secondary completed	193	4.5 [22.7, 26.4]	357	19.5 [12.0, 30.2]	550	21.2 [15.6, 28.3]
Tertiary	105	27.8 [12.5, 50.9]	244	35.1 [18.9, 55.7]	349	32.6 [16.4, 54.3]
Work status						
Unemployed	532	50.4 [39.1, 61.7]	840	45.4 [36.1, 55.1]	1372	47.1 [37.1, 57.4]
Employed	619	49.6 [38.3, 60.9]	1138	54.6 [44.9, 63.9]	1757	52.9 [42.6, 62.9]
Decision-making power						
Low	392	25.1 [19.6, 31.6]	551	12.5 [8.4,18.2]	943	16.8 [11.9, 23.2]
Moderate	463	40.2 [37.9, 42.6]	759	44.2 [39.4, 49.1]	1,222	42.8 [39.5, 46.3]
High	296	34.6 [29.2, 40.5]	668	43.3 [36.9, 50.0]	964	40.2 [34.0, 47.0]
Control of productive assets						
Low	351	27.4 [13.8, 47.0]	496	19.1 [10.5, 32.3]	847	21.9 [11.4, 38.0]
Moderate	340	22.3 [17.1, 28.5]	613	22.8 [13.9, 35.1]	953	22.6 [17.3, 29.1]
High	460	50.3 [38.2, 62.4]	869	58.1 [37.0, 76.5]	1329	55.4 [37.2, 72.3]
Number of ANC visits						
< 4 times	420	25.0 [16.4, 36.2]	324	11.3 [6.1, 19.9]	744	16.0 [9.2, 26.3]
> 4 times	731	75.0 [63.8, 83.6]	1654	88.7 [80.1, 93.9]	2385	84.0 [73.7, 90.8]
Total	1151	34.3 [29.0, 40.0]	1978	65.7 [60.0, 71.0]	3129	100.0

N^^^: unweighted totals; MiP: malaria in pregnancy

### Association between women’s empowerment and uptake of 3+ IPTp-SP

Table 2 presents bivariate (Model I) and multivariate (Model II and III) logistic regression analysis, examining the association between women’s empowerment and the uptake of IPTp-SP. In model I, women with high decision-making power had higher odds of taking 3+ doses of IPTp-SP (cOR = 2.52; CI = 2.06–3.08), as did those with high control over productive assets (cOR = 1.65; CI = 1.45–1.89), tertiary education (cOR = 1.52; CI = 1.14–2.01) and employment (cOR = 1.22; CI = 1.02–1.46) compared to their counterparts with low decision-making power, low control of assets, no schooling/some primary education, and unemployment, respectively.

**Table 2:** Crude and adjusted odds ratios from bivariate and multivariate logistic regression model showing factors associated with optimal uptake of IPTp-SP

	Model I		Model II		Model III	
	COR [95% CI]	P-value	AOR [95% CI]	P-value	AOR [95% CI]	P-value
Decision-making power						
Low^a^	1.00		1.00		1.00	
Moderate	2.21 [1.63, 3.01]	0.000	2.20 [1.54, 3.14]	0.000	2.11 [1.47, 3.04]	0.000
High	2.52 [2.06, 3.08]	0.000	2.25 [1.80, 2.80]	0.000	2.33 [1.81, 3.01]	0.000
Control of productive assets						
Low^a^	1.00		1.00		1.00	
Moderate	1.47 [0.70, 3.05]	0.298	1.45 [0.68, 3.10]	0.323	1.60 [0.76, 3.38]	0.211
High	1.65 [1.45, 1.89]	0.000	1.25 [0.96, 1.64]	0.100	1.20 [0.90, 1.60]	0.210
Level of education						
No schooling or some primary^a^	1.00		1.00		1.00	
Primary completed	1.20 [0.87, 1.66]	0.262	1.29 [0.97, 1.73]	0.081	1.25 [1.00, 1.57]	0.052
Secondary completed	0.96 [0.58, 1.58]	0.860	1.20 [0.61, 2.39]	0.590	1.10 [0.52, 2.34]	0.799
Tertiary	1.52 [1.14, 2.01]	0.005	1.59 [1.18, 2.13]	0.003	1.51 [1.10, 2.06]	0.012
Work status						
Unemployed^a^	1.00		1.00		1.00	
Employed	1.22 [1.02, 1.46]	0.030	0.83 [0.60, 1.14]	0.238	0.80 [0.60, 1.05]	0.107
Knowledge of MiP prevention						
Low^a^			1.00		1.00	
Moderate			0.71 [0.48, 1.04]	0.078	0.72 [0.49, 1.05]	0.086
High			1.14 [0.68, 1.93]	0.611	1.13 [0.70, 1.82]	0.598
Age						
15–24^a^			1.00		1.00	
25–34			0.95 [0.65, 1.38]	0.769	0.88 [0.62, 1.25]	0.475
35–49			1.26 [0.65, 2.44]	0.492	1.15 [0.60, 2.18]	0.670
Marital status						
Not married^a^			1.00		1.00	
Married			0.62 [0.45, 0.87]	0.006	0.65 [0.44, 0.97]	0.038
Gravidity						
One^a^			1.00		1.00	
Two			1.30 [0.86, 1.96]	0.201	1.40 [0.93, 2.12]	0.104
Three			2.83 [0.77, 10.33]	0.113	3.15 [0.97, 10.22]	0.055
Four or more			1.74 [1.15, 2.60]	0.011	2.07 [1.49, 2.87]	0.000
Number of ANC visits						
< 4 times					1.00	
> 4 times					2.80 [2.42, 3.24]	0.000

*COR* crude odds ratio, *AOR *adjusted odds ratio

^a^Reference category

In Model II, after adjusting for individual factors (age, marital status, gravidity and knowledge of MiP prevention; model II), decision-making power and educational attainment remained significantly associated with the uptake of 3+ doses of IPTp-SP (p < 0.05). However, control over assets and employment became less important.

In Model III, which adjusted for the number of ANC visits (the odds of taking 3+ doses of IPTp-SP increased by 2.3 times among women with high decision-making power compared with those with low decision-making power, AOR = 2.33; CI = 1.81–3.01). Similarly, women with moderate decision-making power were more than twice as likely to take 3+ doses of IPTp-SP during their last pregnancy compared to those without decision-making power (AOR = 2.11; CI = 1.47–3.04). Furthermore, after adjusting for individual characteristics and ANC visits, who had attained tertiary education had 50% higher odds of taking 3+ doses of IPTp-SP than those with no schooling or some primary education.

## Discussion

This study examined the association between women’s empowerment and the uptake of 3+ doses of IPTp-SP in two malaria-endemic counties in Kenya. Specifically, this paper explored the association between different dimensions of women’s empowerment (decision-making autonomy, control over productive assets, level of education, and employment status) with optimal uptake of IPTp-SP.

Overall, the study reveals that 75% (751/1151) of women who received less than 3 doses of IPTp-SP despite having attended more than 4 ANC visits for their most recent births. The gap between ANC visits and uptake of 3+ doses of IPTp-SP in the study settings may be attributed to health system-related barriers, such as stock-outs of SP, inadequate capacity of health providers due to lack of training, and non-adherence to national policy guidelines due to competing checklist of ANC elements to be covered [27, 28]. While women will typically accept IPTp-SP if encouraged by a health worker, service delivery barriers, such as workload and long waiting times affect the delivery of quality care [29].

Low uptake of 3+ doses of IPTp-SP may also be due to individual-related factors. For instance, women’s late presentation for ANC delays the start of malaria prevention and treatment in pregnancy and subsequently the number of IPTp doses a woman will receive [13, 14, 30]. The findings underscore the need to address demand- and supply-side barriers to improve the uptake of 3+ doses of IPTp-SP among pregnant women in malaria-endemic regions. Potential interventions could include (1) drawing upon community-based approaches to reduce pressure on and support overburdened health systems, and (2) empowering pregnant women with the appropriate knowledge capacity to make informed decisions on their utilization of IPTp-SP [31].

The analysis of the association between women’s empowerment and IPTp-SP uptake suggests that women’s decision-making power (regarding personal earnings, the respondent’s healthcare, household purchases, and family visits) is an important factor associated with the uptake of 3+ doses of IPTp-SP. The finding is consistent with previous studies on the relationships between women’s decision-making autonomy and the utilization of maternal health services (e.g., ANC, skilled birth, and family planning) [15–18]. One plausible explanation is that women’s empowerment enhances women’s agency, or the ability to define life choices in an evolving social context [21]. Women’s instrumental agency gives them control over maternal health services decisions [32].

Similarly, women who attained tertiary education were more likely to have completed 3+ doses of IPTp-SP compared with their counterparts with no/incomplete primary level of educational attainment. The literature suggests that more educated women may have a greater awareness of the benefits of accessing maternal health services than their less educated counterparts [20]. Additionally, education is likely to improve female autonomy and help women gain greater confidence and ability to make decisions about their own health, including taking 3+ doses of IPTp-SP to prevent malaria during pregnancy. The findings underscore the importance of high maternal education in improving child and maternal health indicators [33, 34].

Including the number of ANC visits in model III had a negligible effect on the association between decision-making power or educational attainment and the uptake of 3+ doses of IPTp-SP. Similar to studies elsewhere [13, 35–37], the number of ANC visits is a powerful predictor of IPTp-SP uptake. However, because the effect of women’s empowerment variables in the regression hardly changed upon introducing the number of ANC visits variable, the results suggest that the influence on IPTp-SP uptake is genuine rather than a proxy for other factors. The strong association between women’s decision-making power and the uptake of 3+ doses of IPTp-SP suggests that interventions designed to empower women may increase their utilization of these services.

The association between control of assets and the uptake of 3+ doses of IPTp-SP was positive but not statistically significant after adjusting for individual characteristics and ANC visits. The study did not also find a statistically significant association between employment (a proxy of economic empowerment) and optimal uptake of IPTp-SP in the adjusted models. However, research shows that women with economic empowerment or cash income have improved access to health services during pregnancy and delivery [38–40]. One potential explanation of the study findings is that financial burden may be a minor barrier to accessing IPTp-SP intervention, which is offered freely as part of ANC services [41].

## Strengths and limitations of the study

The main strength of this study is the use of multidimensional measures of women’s empowerment to examine its association with optimal uptake of IPTp-SP. Women were randomly sampled to participate in the community survey; hence the result can be generalised in similar settings. However, the study has some limitations. The study used cross-sectional data, which does not permit cause-effect relationship analysis. In addition, there is the likelihood of reporting bias/discordance regarding decision-making autonomy within the family due to the subjective nature of this phenomenon. Recall bias on self-reported IPTp-SP use, especially among women who did not have an ANC booklet for verification, may also affect the findings. In addition, the study did not account for unpartnered women when computing decision-making indicators to enable comparison with the partnered ones. Despite these limitations, the findings highlight the association between women’s empowerment and optimal uptake of IPTp-SP in endemic areas in Kenya.

## Conclusion

Women’s decision-making autonomy and educational attainment were positively associated with the uptake of 3+ doses of IPTp-SP. As a result, maternal health interventions should focus on less empowered women, specifically those with less decision-making autonomy and no/low formal education, as they are less likely to achieve optimal uptake of IPTp-SP during pregnancy.

## Data Availability

Data are available upon reasonable request. Requests to access the data may be sent to Population Council, Dataverse, email: publications@popcouncil.org for information on data access.

## Abbreviations

ANC: Antenatal care
DOT: Direct observation therapy
IPTp-SP: Intermittent preventive treatment in pregnancy
KDHS: Kenya Demographic and Health Survey
KIMS: Kenya Malaria Indicator Survey
MiP: Malaria in pregnancy
ODK: Open Data Kit
SP: Sulfadoxine–pyrimethamine
SSA: Sub-Saharan Africa
WHO: World Health Organization
